# Development and Testing of X-Ray Imaging-Enhanced Poly-L-Lactide Bone Screws

**DOI:** 10.1371/journal.pone.0140354

**Published:** 2015-10-14

**Authors:** Wei-Jen Chang, Yu-Hwa Pan, Jy-Jiunn Tzeng, Ting-Lin Wu, Tsorng-Harn Fong, Sheng-Wei Feng, Haw-Ming Huang

**Affiliations:** 1 School of Dentistry, Taipei Medical University, Taipei, Taiwan; 2 Department of General Dentistry, Chang Gung Memorial Hospital, Taipei, Taiwan; 3 Chang Gung University, Taoyuan, Taiwan; 4 Graduate Institute of Biomedical Materials and Tissue Engineering, Taipei Medical University, Taipei, Taiwan; 5 Department of Anatomy, School of Medicine, Taipei Medical University, Taipei, Taiwan; Brandeis University, UNITED STATES

## Abstract

Nanosized iron oxide particles exhibit osteogenic and radiopaque properties. Thus, iron oxide (Fe_3_O_4_) nanoparticles were incorporated into a biodegradable polymer (poly-L-lactic acid, PLLA) to fabricate a composite bone screw. This multifunctional, 3D printable bone screw was detectable on X-ray examination. In this study, mechanical tests including three-point bending and ultimate tensile strength were conducted to evaluate the optimal ratio of iron oxide nanoparticles in the PLLA composite. Both injection molding and 3D printing techniques were used to fabricate the PLLA bone screws with and without the iron oxide nanoparticles. The fabricated screws were implanted into the femoral condyles of New Zealand White rabbits. Bone blocks containing the PLLA screws were resected 2 and 4 weeks after surgery. Histologic examination of the surrounding bone and the radiopacity of the iron-oxide-containing PLLA screws were evaluated. Our results indicated that addition of iron oxide nanoparticles at 30% significantly decreased the ultimate tensile stress properties of the PLLA screws. The screws with 20% iron oxide exhibited strong radiopacity compared to the screws fabricated without the iron oxide nanoparticles. Four weeks after surgery, the average bone volume of the iron oxide PLLA composite screws was significantly greater than that of PLLA screws without iron oxide. These findings suggested that biodegradable and X-ray detectable PLLA bone screws can be produced by incorporation of 20% iron oxide nanoparticles. Furthermore, these screws had significantly greater osteogenic capability than the PLLA screws without iron oxide.

## Introduction

The main disadvantage of metallic bone implants and hardware is that they require an additional removal operation after healing. Biodegradable polymers were developed to overcome this drawback [1–2]. The visibility of these polymer implants in bone is crucial to orthopedic surgeons to ensure accurate placement of the implants during surgery. In addition, biodegradable implants require radiopacity to facilitate the evaluation of their degradation during healing. However, native biodegradable polymers cannot be detected by commonly used X-radiography techniques because they exhibit low specific gravity and electron density, which results in low X-ray absorption [3].

Increasing the specific gravity of a polymer by incorporating heavy elements is the primary strategy for improving radiopacity [3]. Among the radiopaque polymers, iodine is the most commonly used element to enhance the contrast of a polymer because it can be incorporated into various polymers. For example, it has been used to fabricate radiopaque polyurethanes [3], polyester [4], and polymethylmethacrylate [5]. In addition to iodine, researchers also used barium sulfate and bismuth bromide to produce radiopaque polyurethanes [6–7]. However, these additives had adverse effects on the mechanical properties of the polymer matrix [3,5]. Furthermore, they resulted in degradation of the polymer and leaching of the contrast agent into the surrounding tissue, which induced adverse effect [4,7]. For example, leaching of barium sulfate from bone cement triggered osteolysis at the bone/implant interface [5]. Investigators then developed radiopaque polymers without incorporation of radiopaque additives [7]. The strategy of this technique was to use iodine-containing molecules such as triiodobenzoic acid to bind covalently to the polymer backbone [3–4,8]. However, these materials exhibited low molecular weights and poor precipitation properties, and thus less than the desired radiopacity [4].

Polylactic acid (PLA) is one of the biodegradable polymers with widespread application in orthopedic devices, dental rehabilitation, drug delivery, and injectable tissue engineering [8]. Additionally, PLA is one of the 3D printing materials used in bone tissue engineering [9], which is attracting considerable attention as the use of 3D printing technologies advances. Nonetheless, studies of radiopaque PLA polymers are rare.

In 2015, Lei developed an injectable thermogel with strong X-ray opacity that incorporated 2,3,5-triiodobenzoic acid on the hydrophobic end of the poly(ethylene glycol) (PEG)-PLA copolymer. However, the biocompatibility of the leached contrast agent during PEG-PLA degradation was not evaluated [8]. In 2006, Torrente used iron oxide (Fe_3_O_4_) nanoparticles to label human mesenchymal stem cells and seeded the cells on PLA scaffolds [10]. Their results indicated that Fe_3_O_4_ nanoparticle labeling was a promising approach for visualizing the cells using micro-CT. Fe_3_O_4_ nanoparticles not only showed strong contrast on magnetic resonance imaging [11], but exhibited excellent osteogenic effects as well [12–14].

Recently, investigators integrated Fe_3_O_4_ nanoparticles into poly-L-lactic acid (PLLA) to fabricate multifunctional biomaterials [15–17]. However, whether or not the Fe_3_O_4_ nanoparticle/PLLA scaffold provided radiopacity was not systematically studied. The purpose of this study was to fabricate biodegradable bone screws using an Fe_3_O_4_ nanoparticle/PLLA polymer and test whether or not they exhibited X-ray opacity. In addition, the CT visibility and osteogenic effects of the bone screws were evaluated.

## Materials and Methods

### Preparation of Fe_3_O_4_ nanoparticle/PLLA composites

The fabrication procedure of the Fe_3_O_4_ nanoparticle (nano-Fe_3_O_4_)/PLLA composite, including compounding, pelleting, drying, and injection molding was performed as in previous studies [16]. Briefly, the Fe_3_O_4_ nanoparticles (99.9%, 50 nm, Long Ton, Inc., Taipei, Taiwan) and PLLA powder (molecular weight of 100 kDa, Wei Mon Industry Co., Taipei, Taiwan) were dried at 80°C for 24 hours. The two materials were then mixed using a twin-screw extruder at a mixing temperature of 150°C. The extruded composite strands were cooled in a water bath at a constant temperature of 25°C. Then the composite strands were cut into small granules with a pelletizer and dried overnight. In this study, four nano-Fe_3_O_4_/PLLA composites with Fe_3_O_4_ proportions of 0%, 20%, 30%, and 40% (w/w) were prepared. The samples were injection molded and tested for strength. For the three-point bending tests, rectangular plates 12 × 6.3 × 2.1 mm were produced by injection molding. For tensile strength testing, the Fe_3_O_4_/PLLA granules were injection molded into I-shaped testing plates according to the ASTM (American Society for Testing and Materials) D638-V standard [18]. For preparing materials for 3D printing, nano-Fe_3_O_4_/PLLA strands 1.65mm in diameter and 20 cm in length were also produced by injection molding.

### Mechanical testing

To detect the flexural strength of the fabricated nano-Fe_3_O_4_/PLLA plates, three-point bending tests were performed using a universal testing machine (AGS-1000D, Shimadzu, Tokyo, Japan) according to a previously described method [16]. Before testing, the specimen was placed on two supports 12 mm apart. A vertical force, provided by a load cell, was applied at the middle position of the two supports with a displacement control mode. The crosshead speed was set at a rate of 10 mm/min. The maximum flexural strength of the sample was recorded immediately before fracture.

Ultimate tensile strength is the maximum stress that a material can withstand while being stretched or pulled. It is a basic property of materials for evaluating the strength of the material when it is subjected to a load. The ultimate tensile strength of the fabricated nano-Fe_3_O_4_/PLLA was tested according to the ASTM D638-V standard. The injection molded sample was fixed by a pair of holding devices at the upper and lower part of the plate. The test sample was aligned in the grips of the universal testing machine. Before mechanical testing, a pre-load was directly applied to the testing screws. The procedure was conducted at a set displacement control mode. A vertical load was applied at a rate of 1 mm/min and the ultimate tensile strength of the fabricated nano-Fe_3_O_4_/PLLA was recorded when the sample fractured.

The tensile strength is determined by dividing the peak load by the cross-sectional area of the narrow region of the I-shape specimen. Five samples were tested in each experimental group. All data from the mechanical tests are presented as means ± standard deviations. Differences in mechanical strength between the neat PLLA screws and the nano-Fe_3_O_4_/PLLA screws were tested using one-way analysis of variance. Probability values less than 0.05 were considered significant.

### Fabrication of nano-Fe3O4/PLLA bone screw

The nano-Fe_3_O_4_/PLLA bone screws were fabricated using a fused deposition modeling technique. It is one of the techniques used for 3D print modeling, prototyping, and production applications. The bone screws were 3D printed using a commercially available machine (Born One, Wanwall, Honk Kong, China) with a resolution of 0.15 mm. The extrusion temperature was set at 185°C. The screws were designed as a self-tapping screw with a geometric size of 3.1 mm in diameter and 12 mm in length. During the above mechanical tests, we found that a nano-Fe_3_O_4_/PLLA ratio greater than 20% significantly decreased the mechanical strength of the PLLA. In addition, when the nano-Fe_3_O_4_ content was 30% or higher, the material could not be extruded from the extrusion nozzle head of the 3D printing machine. Thus, the nano-Fe_3_O_4_/PLLA composite chosen to make the bone screws for the in situ experiments was 20% (w/w).

Injection molded screws with the same diameter and geometric thread size as the tested screws were produced to assess the healing process in situ. For both injection molded and 3D printed screws, neat PLLA screws were fabricated and used in the control group.

### Experimental animals and implant surgery

The animal study was approved by the Institutional Animal Care and Use Committee of Taipei Medical University, Taipei, Taiwan (LAC-2014-0123). Twelve New Zealand white rabbits (average age, 8 months) weighing 3.0–3.5 kg served as the subjects. The flat medial femoral condyles of both legs were used as the surgical sites. The bone screw operations were performed under sterile conditions. Tiletamine-zolazepam (15 mg/kg) (Zoletil 50, Virbac, Carros Cedex, France) was given by intramuscular injection for general anesthesia. The surgical sites were shaved and disinfected using an iodine solution. Local anesthesia was achieved by injecting 2% epinephrine (1.8 ml) at the femoral condyles. After skin incision and muscle dissection, the bone surfaces at the medial aspect of distal femoral condyle were exposed using a periosteal elevator. The bone screw implant sites were prepared by drilling cavities with the same diameter as the bone screw. Normal saline solution was used to cool the drill during the entire surgical procedure. The bone screws were then implanted, and the muscle and skin were closed in layers with absorbable sutures (Vicryl^®^ 4.0, Ethicon, Somerville, NJ, USA). Postoperative antibiotics and analgesics were administered intramuscularly for 3 days to control pain and reduce infection risk.

The observation time points were set at 2 and 4 week after surgery. A total of 24 screws were made using the two fabrication methods (12 by injection-molding and 12 by 3D-printing). Six of the screws in each fabrication groups were neat PLLA (control) and six were 20% nano-Fe_3_O_4_/PLLA. For each observed time point, screws were implanted into the femoral condyles of both legs of the 6 rabbits (three control screws in one leg and three nano-Fe_3_O_4_/PLLA screws in the other for each of the fabrication methods). All data from the animal experiments are presented as means ± standard deviations. Student’s *t*-test was used to examine the differences in bone volumes between the rabbits implanted with the neat PLLA screws and the 20% nano-Fe_3_O_4_/PLLA screws for each fabrication method. Probability values less than 0.05 were considered significant.

### Histology examination and radiopacity tests

The rabbits were sacrifed after 2 and 4 weeks of healing using a human method to minimize pain and suffering. Briefly, thsy were killed under general anesthesia by bilateral carotid perfusion with a solution of 37% formalin and 99% methanol in distilled water (1:1.5 v/v) following an intravenous injection of heparin to prevent intravascular clotting. The femoral condyles containing the bone screws were resected and placed in a fixative solution consisting of 10% formalin. Then, the screw/bone samples were scanned using a micro computed tomographic (micro-CT) scanner (Skyscan 1076, Skyscan, Antwerp, Belgium) at an energy level of 55 kV at 181 A. The pixel resolution was 18 μm using a 0.5-mm aluminum filter. The 3D images were reconstructed using commercial software (CTAn, Skyscan, Antwerp, Belgium). Bone healing was normalized by calculating the bone volume from the top surface of the cortical bone to a depth of 1.1 mm. At this position, a particular area with a diameter of 4.97 mm around the implant was selected for the calculation.

The screw/bone samples were used for histomorphometric evaluation after micro-CT. After demineralization [19], the screw/bone samples were sectioned into 2-mm slices through the long axis of the screws with a diamond-edged saw. The screw was removed prior to section. After dehydration, the specimens were embedded in paraffin wax. An ultramicrotome (Bright 5040, Bright Instrument, Cambs, England) was used to slice the embedded samples into 10-μm sections. Then, the specimens were stained with hematoxylin and eosin as described previously [20]. For histological examination, a light microscope (CH2, Nikon, Tokyo, Japan) equipped with a digital camera (Coolpix 950, Nikon) was used for digitizing the histological images.

## Results

**Fig 1:**
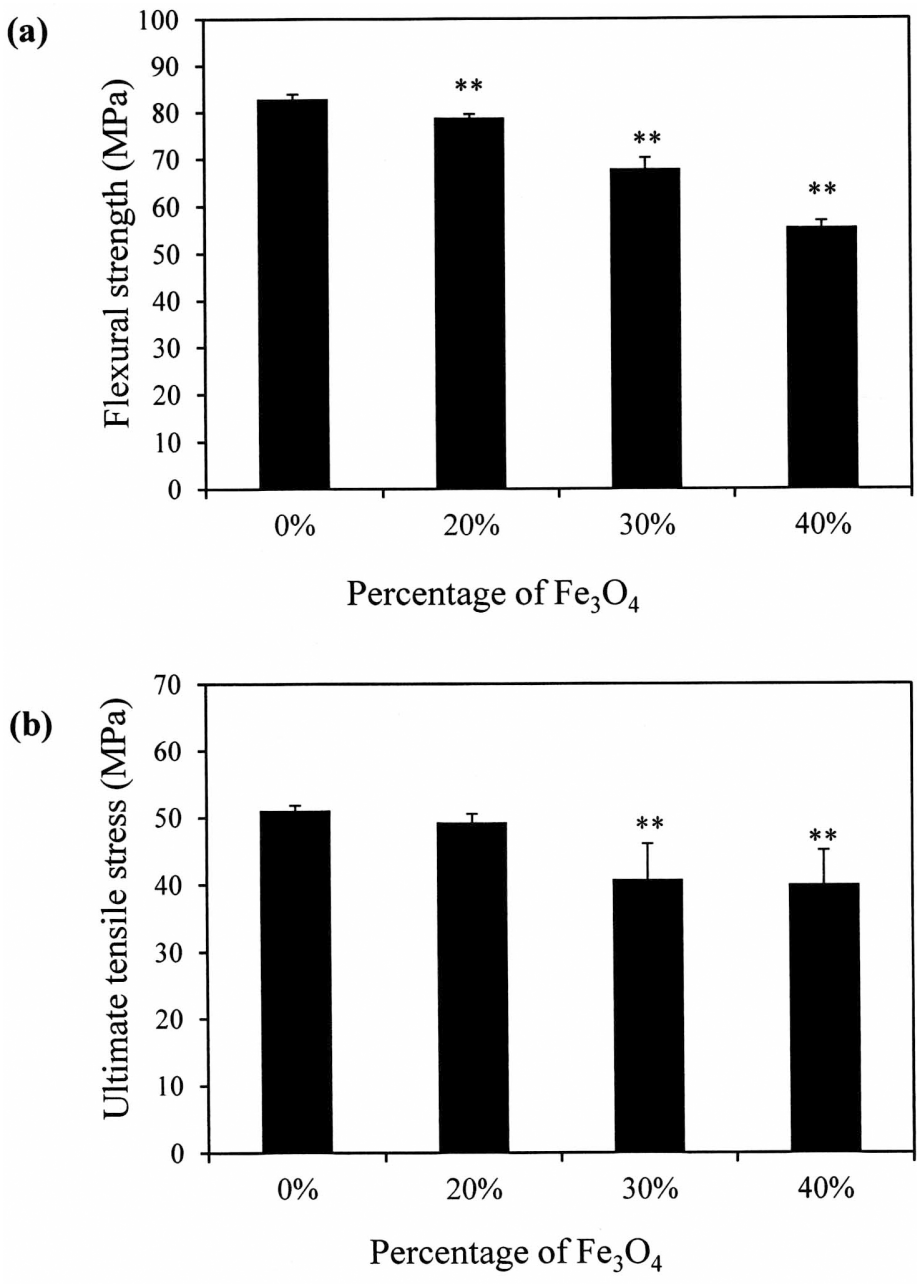
Flexural strength (a) and ultimate tensile stress (b) data for the nano-Fe_3_O_4_/poly-L-lactide (PLLA) composites with various proportions of nano-Fe_3_O_4_ particles. The addition of 20% nano-Fe_3_O_4_ to the PLLA had no significant effect on the ultimate tensile strength of the PLLA composites. **p < 0.01.

In Fig 1a, the flexural strength of the neat PLLA polymer is 82.9 ± 0.9 MPa. The addition of 20% nano-Fe_3_O_4_ to the PLLA had slightly reduced the flexural strength of the PLLA polymer. However, the flexural strengths significantly decreased to 67.9 ± 2.3 and 55.57 ± 1.4, respectively, when the nano-Fe_3_O_4_ contents were increased to 30% and 40% (p < 0.01). The ultimate tensile strength of the neat PLLA is 51.1 ± 0.7 MPa (Fig 1b). The statistical analysis showed no difference in the ultimate tensile strength when the Fe_3_O_4_ concentration was 20%. However, when the nano-Fe_3_O_4_ was 30% or higher, a dramatic adverse effect on ultimate tensile strength was observed.

**Fig 2:**
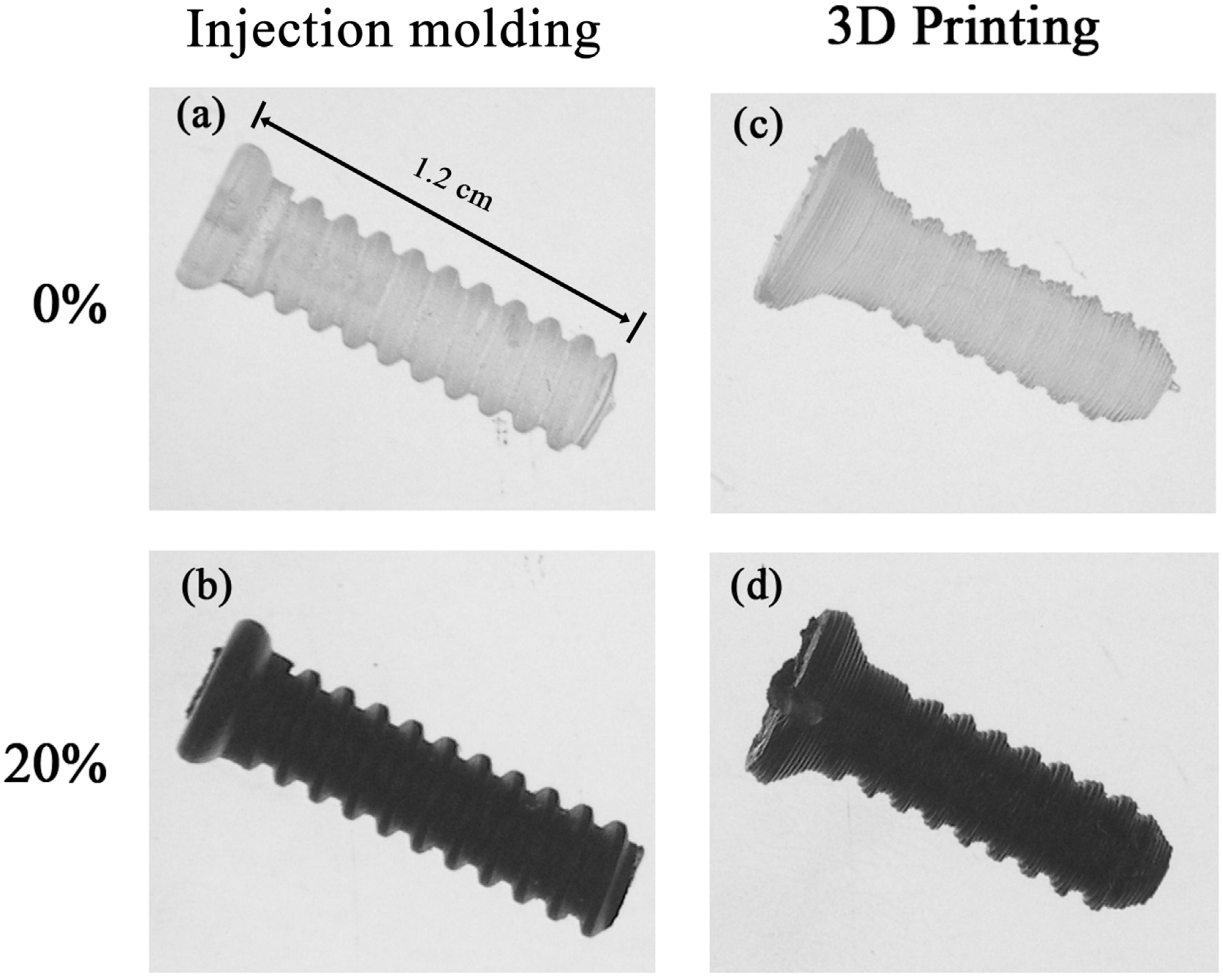
Images of the four fabricated poly-L-lactide (PLLA) bone screws. The screws containing 0% (a and c) and 20% (w/w) Fe_3_O_4_ nanoparticles (b and d). Injection molding (a and b) and 3D printing (c and d) methods were used to produce the screws.

Fig 2 shows the bone screws fabricated by injection molding and 3D printing. When the nano-Fe_3_O_4_ content was 30%, the material could not be extruded from the extrusion nozzle head of the 3D printing machine. Accordingly, 20% nano-Fe_3_O_4_/PLLA was chosen for fabricating the bones screws for the animal experiments.

**Fig 3:**
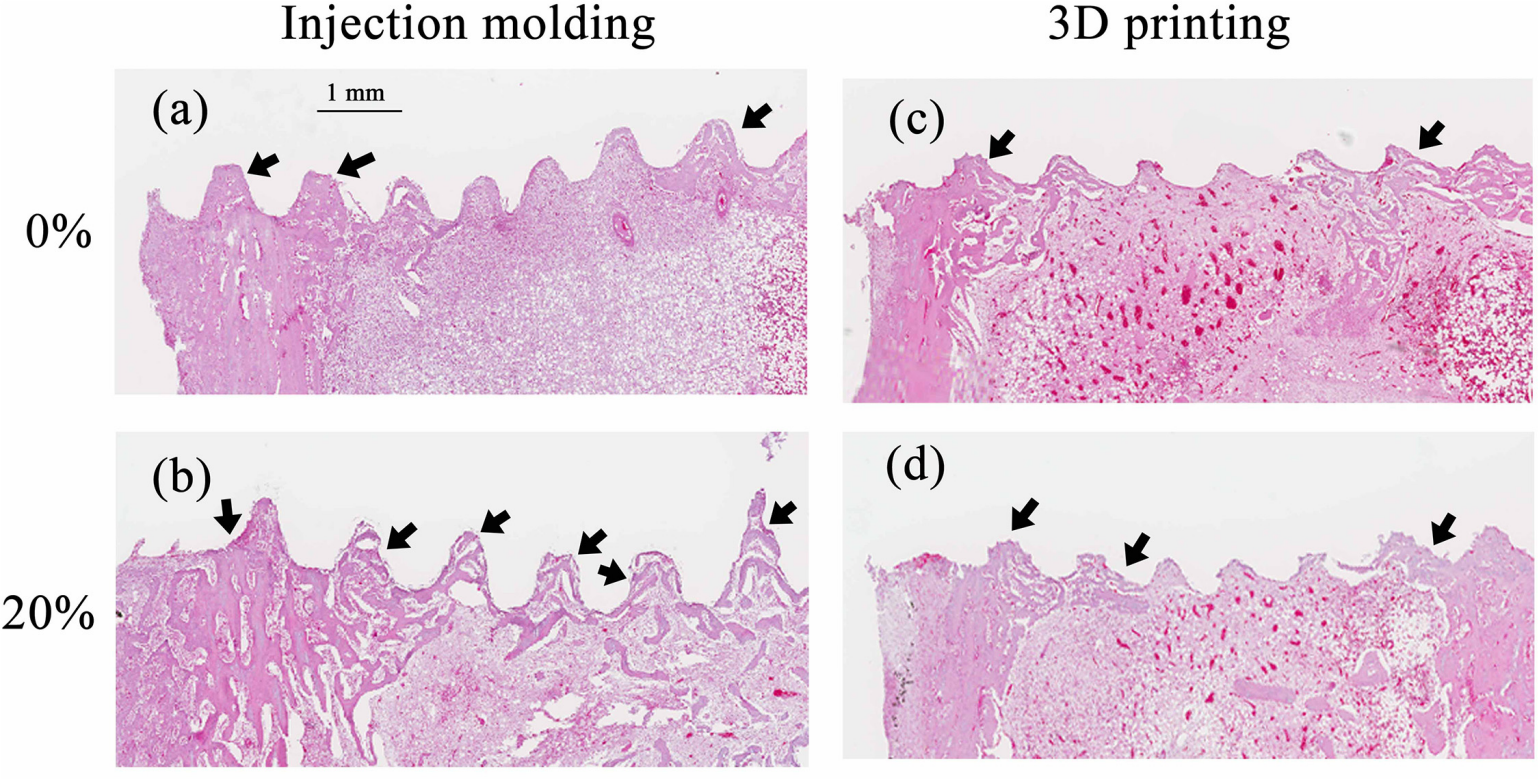
Histologic examination of bone tissue at the screw/bone interface 2 weeks after implanting the screws. Neat poly-L-lactide (PLLA) screws were fabricated by injection molding (a) and 3D printing (c) methods. Screws made of PLLA mixed with 20% Fe_3_O_4_ nanoparticles fabricated by injection molding (b) and 3D printing (d). New bone (black arrows) was visible between more of the threads and occupied a greater area with the 20% nano-Fe_3_O_4_/PLLA screw compared to the neat PLLA screw. Scale bar, 1.0 mm.

**Fig 4:**
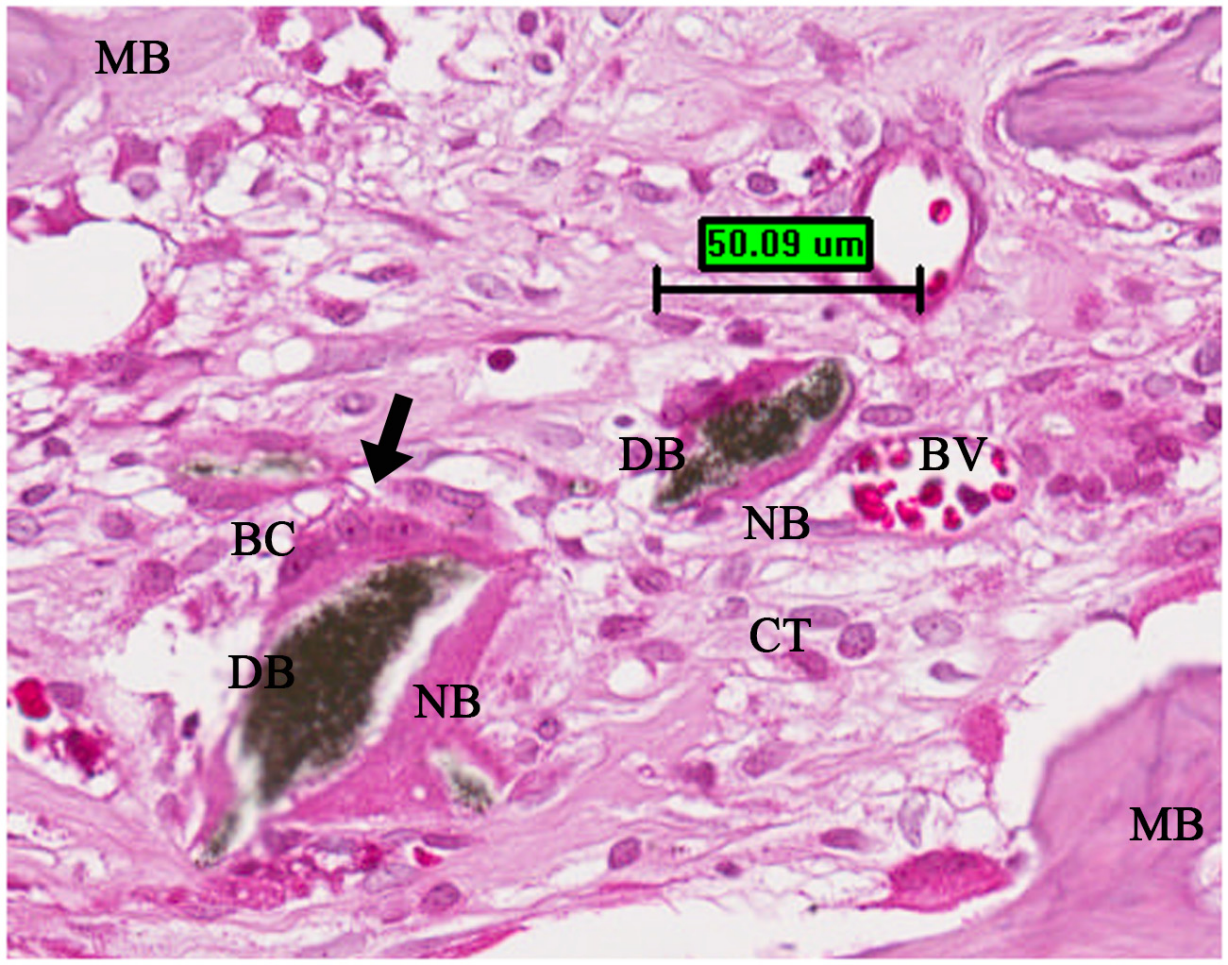
A typical example of the histologic image at the screw/bone interface 4 weeks after implanting the screws. The leached debris of the nano-Fe_3_O_4_/poly-L-lactide (PLLA) composite was surrounded by newly formed bone and bone cells (black arrow). BC: bone cell; BV: blood vessel; CT: connective tissue; DB: leached debris; MB: mature bone; NB: new bone. Scale bar, 50 μm.

The histologic analysis of the screw/bone samples at 2 weeks after implantation was shown in Fig 3. No inflammatory response and adverse effect on bone tissue were observed in the threaded implant site. Additionally, noticeable new bone had formed at the screw/bone interfaces of the cortical bone. For both injection molded and 3D printed samples new bone was visible between more of the threads and occupied a greater area with the nano-Fe3O4/PLLA screw compared to the neat PLLA screw. After 4 weeks of healing, leached nano-Fe_3_O_4_/PLLA debris surrounded by new bone was found at the bone/screw interface (Fig 4). Bone cells not only can be found at the screw/bone interface, but also on the surface of the debris.

**Fig 5:**
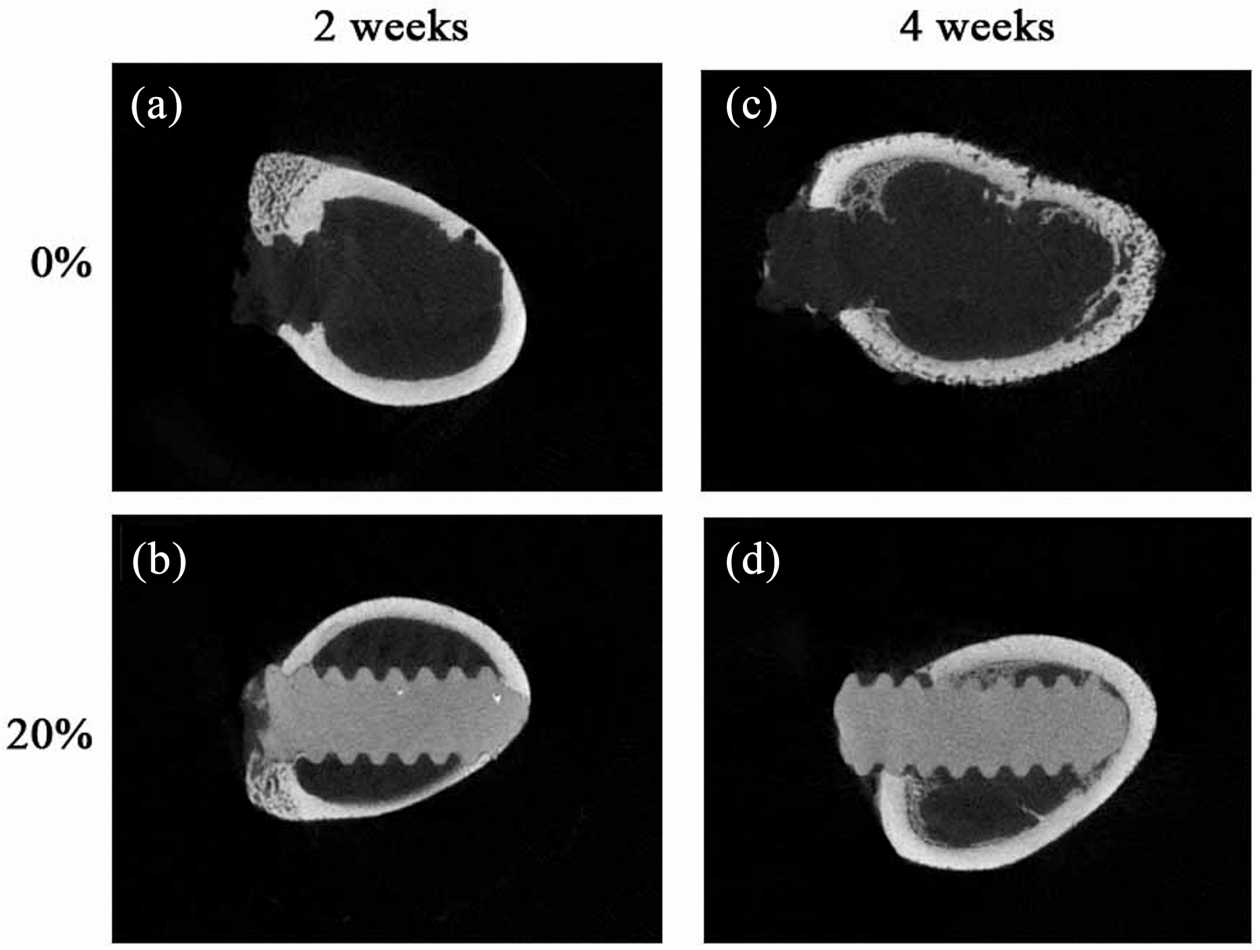
Micro computed tomographic images of injection molded PLLA screws implanted in rabbit bone. The boundary and locations of the neat PLLA screws cannot be distinguished from surrounding tissues after 2 (a) and 4 (c) weeks of healing. With the addition of 20% Fe_3_O_4_ nanoparticles to the PLLA, the screws exhibited a significant improvement in radiopacity for clinical visibility after 2 (b) and 4 (d) weeks of healing.

**Fig 6:**
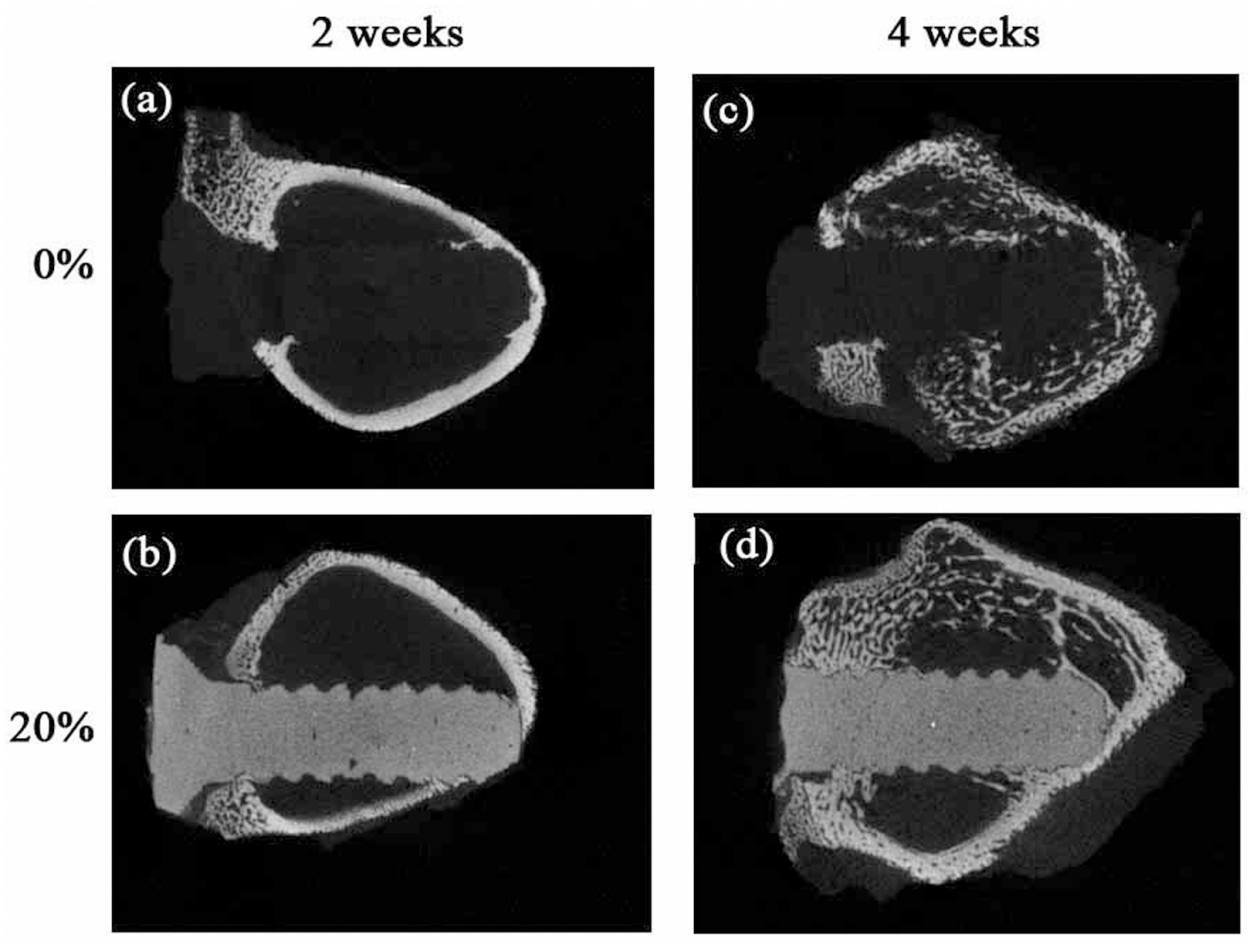
The micro computed tomographic images of the 3D printed PLLA screws implanted in rabbit bone. The boundary and locations of the neat PLLA screws cannot be distinguished from surrounding tissues after 2 (a) and 4 (c) weeks of healing. With the addition of 20% Fe_3_O_4_ nanoparticles to the PLLA, the screws exhibited a significant improvement in radiopacity for clinical visibility after 2 (b) and 4 (d) weeks of healing.

To test the radiopacity of the the nano-Fe_3_O_4_/PLLA screw, the screw/bone samples were scanned using a micro computed tomographic (micro-CT) scanner. Fig 5 shows typical micro-CT images of bone specimens implanted with injection molded screws after 2 and 4 weeks of healing. The radiopacity of the neat PLLA screws was very close to that of the surrounding soft tissues (Fig 5a and 5c). It is hard to discern the boundary and location of the neat PLLA screw from the surrounding tissue. In contrast, the PLLA screws mixed with 20% nano-Fe_3_O_4_ particles exhibited strong radiopacity (Fig 5b and 5d) compared to the screw fabricated with neat PLLA. It was easy to distinguish the boundary of the 20% nano-Fe_3_O_4_ PLLA screw from the surrounding hard tissues. Similar results were also observed for the 3D printed samples (Fig 6). The radiopacity of the nano-Fe_3_O_4_ particles is much greater than that of the PLLA and surrounding soft tissue.

**Fig 7:**
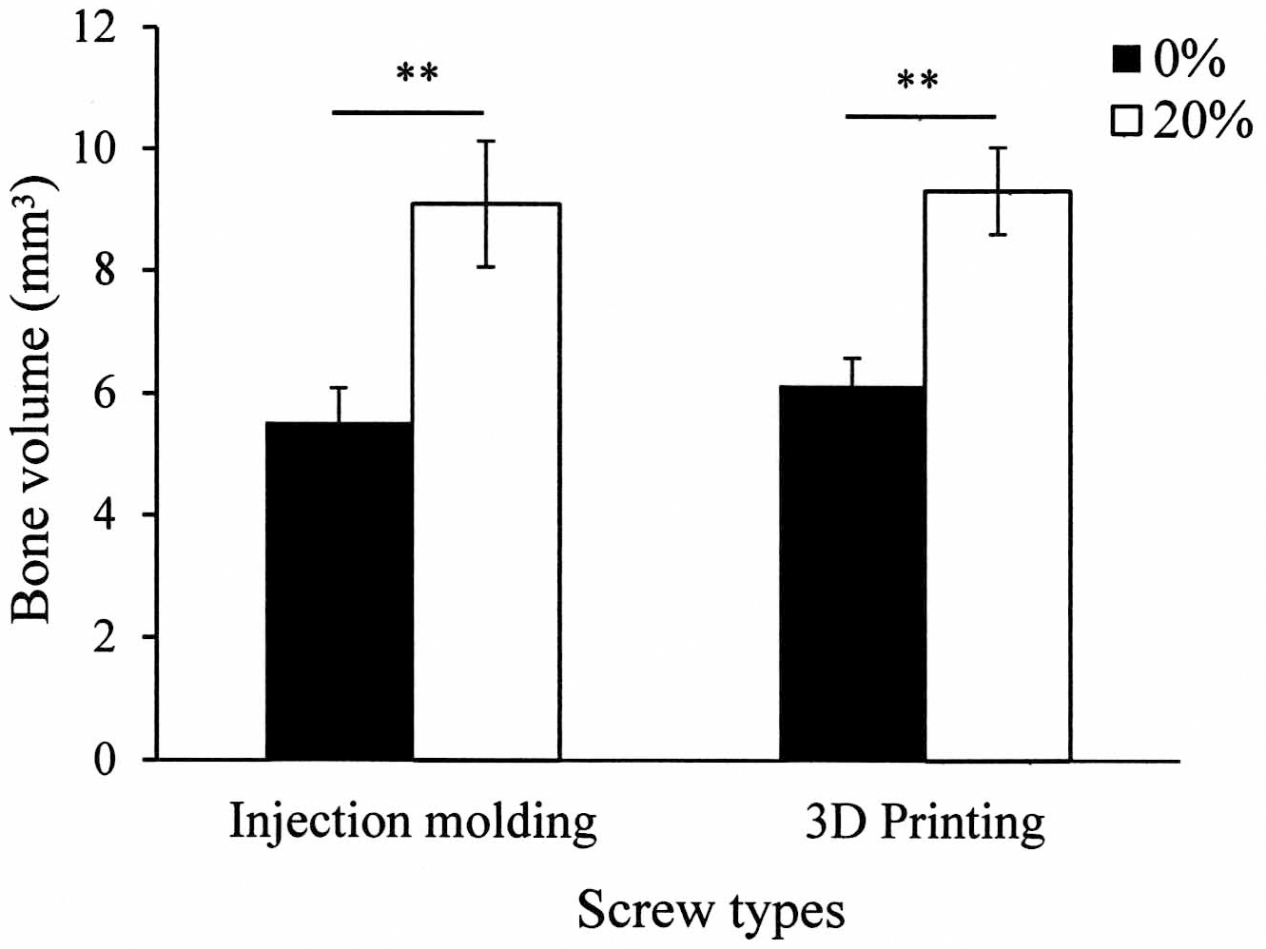
Bone volumes calculated from the micro computed tomographic images for the injection molded and 3D printed fabrication methods. The bone volumes surrounding the screws increased when the screws are fabricated with 20% nano-Fe_3_O_4_/PLLA. (**p < 0.01)

The average values of bone volume after 4 weeks of healing were analyzed on the micro-CT images. Fig 7 shows that the bone volumes surrounding the neat PLLA screws are 5.5 ± 0.5 mm^3^ and 6.1 ± 1.0 mm^3^ for the injection molded and 3D printed screws, respectively. These values are increased to 9.1 ± 0.4 mm^3^ and 9.3 ± 0.7 mm^3^, respectively, when the screws are fabricated with 20% nano-Fe_3_O_4_/PLLA. The statistical analysis revealed that this increase was significant (*p* < 0.01).

## Discussion

X-ray CT was used for *in vivo* detection and morphologic analysis of the implanted devices and materials [8]. As shown in Figs 5 and 6, the neat PLLA screws lacked radiopacity making their position and degradation status difficult to evaluate during radiography. Although addition of contrast agents make polymeric devices radiopaque, these compounds reduce the mechanical and thermal stability of the polymer [3–5,7]. The radiopaque additives affect the crystallinity and density of the polymer, which may result in side effects from implanted highly crystalline devices [18]. Accordingly, we sought the optimal ratio of the contrast agent and polymer to provide sufficient X-ray imaging enhancement without degrading the mechanical properties of the polymer matrix. Based on previous reports, the optimal ratio of contrast agent should be between 20% and 30%. Several investigators reported that 20% contrast agent was necessary to provide sufficient radiopacity in the polymer for biomedical applications [4,7]. Coutu et al. (2013) fabricated a new radiopaque embolizing agent that enhanced the X-ray opacity of chitosan. They found that the addition of 20% contrast agent to the polymer provided optimal X-ray visibility and maintained the physical and mechanical properties of the composite polymer [21]. In our study, Fig 1b shows that the ultimate tensile strength of the neat PLLA is 51.1 MPa. This value is close to that of a previous report in which the ultimate tensile strength of the PLLA manufactured by melting process was approximately 60 MPa [18]. Our results also showed that the addition of nano-Fe_3_O_4_ particles at percentages greater than 20% significantly reduced the ultimate tensile stress of the PLLA.

In bone tissue engineering, biomaterial scaffold was used to repair large-scale bone defects. The application of 3D printed scaffolds is becoming popular due to this method can directly print designed scaffolds with complex shape. Since nano-Fe_3_O_4_/PLLA bone screws can be fabricated by 3D printing (Fig 2), the same protocol can also be used for 3D printed scaffolds with designed shapes. However, the 3D printing technique used in this study was a fused deposition modeling method. The disadvantage of this technique is that the production process is time-consuming and the fabricated screws lack the fine thread architecture and smooth surface of injection molded screws (Fig 2). In addition, the screw samples were printed layer-by-layer. Thus the mechanical property of these 3D printed samples varies depending on the loading direction with respect to the build direction. With this regard, the build direction should be taken into consideration when the scaffolds and implant devices were fabricated using current method.

During fused deposition modeling, the samples are produced by extruding the composite material via a heated extrusion nozzle in the print head. Accordingly, the material used for this method must be a thermoplastic material with appropriate viscosity when melted. Although it is reported that 3D printing can directly print PLA devices with complex shapes [9,18], the addition of nano-Fe_3_O_4_ particles to the PLLA affects its viscosity. Lei et al. (2015) reported that an abrupt increase in the viscosity of the radiopaque thermal gel was observed when it was mixed with the contrast agent at concentrations higher than 30% [8]. We also found that the PLLA mixed with the contrast agent at concentrations greater than 20% could not be extruded from the extrusion nozzle head of the 3D printer. Based on these findings, combined with the data shown in Fig 1, we discovered that 20% was the optimal percentage of nano-Fe_3_O_4_ particles for incorporation into the PLLA to produce visible PLLA radiopacity. Thus, we fabricated our radiopaque, biodegradable bone screws with 20% (w/w) nano-Fe_3_O_4_. Since several factors, such as nanoparticle size, affect the viscoelastic property of PLLA composite, the optimum loading percentage should be tested when the other nanoparticles being used.

The primary advantage of using PLLA for an implantable device is that its degradation in situ is nontoxic. PLLA is naturally metabolized to carbon dioxide and water [22]. Nonetheless, when PLLA is mixed with contrast agents, the biocompatibility of these additives becomes a concern. To enhance the radiopacity of bone cement, several investigators used BaSO_4_ as the contrast agent. However, leaching of the BaSO_4_ induced osteolysis [5]. In Figs 5 and 6 (c and d), both injection molded and 3D printed nano-Fe_3_O_4_/PLLA screws show no local osteolysis during the healing period. Interestingly, quantification of CT images demonstrated that PLLA screws fabricated with 20% Fe_3_O_4_ nanoparticles exhibited 1.5-fold higher bone volume compared to the neat PLLA screws (Fig 7). As shown in Fig 4, bone cells are not only found at the implant/bone interface but also on the surfaces of leached nano-Fe_3_O_4_/PLLA debris. Previous reports indicate that Fe_3_O_4_ nanoparticles have an osteogenic effect on bone cells [12–14]. Thus, the PLLA screws fabricated with 20% nano-Fe_3_O_4_ particles exhibited higher bone volume at the implant/bone interface (Fig 7) than the neat PLLA screws can contribe to the addition of Fe_3_O_4_ nanoparticles to the PLLA.

## Conclusions

In conclusion, a novel radiopaque, biodegradable, and 3D printable bone screw was fabricated in this study for the first time. The PLLA composite containing 20% nano-Fe_3_O_4_ particles exhibited excellent radiopacity in the *in vivo* tests. Additionally, the nano-Fe_3_O_4_ particles show potential as a catalyst of osteogenesis during the PLLA degradation process.
